# T-cell contact-dependent regulation of CC and CXC chemokine production in monocytes through differential involvement of NFκB: implications for rheumatoid arthritis

**DOI:** 10.1186/ar2077

**Published:** 2006-11-13

**Authors:** Jonathan T Beech, Evangelos Andreakos, Cathleen J Ciesielski, Patricia Green, Brian MJ Foxwell, Fionula M Brennan

**Affiliations:** 1 Kennedy Institute of Rheumatology Division, Imperial College School of Medicine, 1 Aspenlea Road, Hammersmith, London W6 8LH, UK; 2 Foundation for Biomedical Research of the Academy of Athens, Center for Immunology and Transplantations, 4 Soranou tou Ephessiou, 11527 Athens, Greece

## Abstract

We and others have reported that rheumatoid arthritis (RA) synovial T cells can activate human monocytes/macrophages in a contact-dependent manner to induce the expression of inflammatory cytokines, including tumour necrosis factor alpha (TNFα). In the present study we demonstrate that RA synovial T cells without further activation can also induce monocyte CC and CXC chemokine production in a contact-dependent manner. The transcription factor NFκB is differentially involved in this process as CXC chemokines but not CC chemokines are inhibited after overexpression of IκBα, the natural inhibitor of NFκB. This effector function of RA synovial T cells is also shared by T cells activated with a cytokine cocktail containing IL-2, IL-6 and TNFα, but not T cells activated by anti-CD3 cross-linking that mimics TCR engagement. This study demonstrates for the first time that RA synovial T cells as well as cytokine-activated T cells are able to induce monocyte chemokine production in a contact-dependent manner and through NFκB-dependent and NFκB-independent mechanisms, in a process influenced by the phosphatidyl-inositol-3-kinase pathway. Moreover, this study provides further evidence that cytokine-activated T cells share aspects of their effector function with RA synovial T cells and that their targeting in the clinic has therapeutic potential.

## Introduction

A large and diverse range of proinflammatory cytokines and chemokines have been detected in the synovium of patients with rheumatoid arthritis (RA) (reviewed in [1,2]). This diversity is not surprising, considering the heterogeneous mixture of activated cells found at the sites of inflammation of RA synovium, which include macrophages, T cells, endothelial cells, fibroblasts and plasma cells.

Of particular interest are chemokines, which selectively recruit haemopoietic cells from the blood into the inflamed synovium. Several chemokines have been detected in RA synovium and include IL-8 (CXCL8) [3], monocyte chemoattractant protein 1 (MCP-1; CCL2) [4], epithelial neutrophil activating peptide 78 [5], macrophage inflammatory protein 1 alpha (MIP-1α; CCL3) [6], macrophage inflammatory protein 1 beta (MIP-1β; CCL4) [7], RANTES (CCL5) [7] and growth-related gene product alpha (GROα; CXCL1) [8] (reviewed in [9]). What regulates chemokine gene expression in the RA synovium, however, remains to be determined.

T cells were recently shown to be essential for the production of proinflammatory cytokines from macrophages in RA synovial tissue [23]. Although synovial CD4^+ ^T cells proliferate poorly and produce low levels of IL-2 and interferon gamma [10-12], they express cytokines and activation markers [13] – and when put in contact with synovial fibroblasts or monocytes/macrophages, synovial CD4^+ ^T cells induce high levels of inflammatory cytokines [14-16].

*In vitro*, we have shown that T cells activated by an anti-CD3 cross-linking antibody (that mimics TCR engagement (Ttcr)) or stimulated with a 'cocktail' of cytokines (designated cytokine-activated T cells (Tck)) also stimulate monocytes in a contact-dependent manner to produce cytokines that include IL-1β, TNFα, IL-12, IL-6 and IL-10 [15,17-21]. While the molecules involved in this process have not been fully defined, a number of T-cell-associated cell surface receptors/ligands, including CD69 [17], CD40L [18], CD11b and CD2, have been suggested of importance.

Histologically, T cells are often found in close contact with macrophages in the interstitium of RA synovial tissue [22] and T-cell depletion rapidly diminishes macrophage TNFα synthesis in RA synovial cultures [23].

We previously reported that the contact-dependent effector function of RA T cells in the joint is identical to that displayed by bystander-activated T cells (Tck), which can be expanded from normal blood with a cytokine cocktail containing TNFα, IL-6 and IL-2 over an 8-day period [21,23]. RA synovial T cells and Tck cells both induce TNFα production in resting monocytes in a cell-contact dependent manner, which is abrogated by blockade of the transcription factor NFκB but is augmented if phosphatidyl-inositol-3-kinase (PI3K) is inhibited. Normal blood T cells activated 'conventionally' via the TCR with cross-linked anti-CD3 antibody result in TNFα production from monocytes that is unaffected by NFκB blockade, but is inhibited in the presence of PI3K blocking drugs [23].

In the present report we investigated whether chemokine production from macrophages can also be induced in a contact-dependent manner by activated blood T cells, or indeed by T cells freshly isolated from rheumatoid tissue. We also examined which signalling pathways in macrophages are rate-limiting for the expression of chemokines after T-cell contact, with particular reference to the transcription factor NFκB, in order to gain insight into the regulation of chemokines at sites of inflammation.

## Materials and methods

### Isolation of peripheral blood monocytes and lymphocytes

Human monocytes were isolated from single-donor platelet pheresis residues purchased from the North London Blood Transfusion Service (Colindale, UK). Mononuclear cells were isolated by Ficoll/Hypaque centrifugation (specific density 1.077 g/ml; Nycomed Pharma A.S., Oslo, Norway), prior to cell separation in a Beckman JE6 elutriator (Torrence, CA, USA). Elutriation was performed in culture medium containing 1% heat-inactivated FCS. The monocyte purity and lymphocyte purity were assessed by flow cytometry, and fractions were typically >80% and 90% pure, respectively.

### T-cell stimulation and fixation

Elutriation-enriched lymphocytes were resuspended in RPMI 1640 (containing 10% heat-inactivated AB^+ ^human serum; (Biowittaker, Wokingham, UK) at 1 × 10^6 ^cells/ml. The resuspended lymphocytes were then cultured in six-well cluster culture plates (Falcon, Bedford, MA, USA) at 37°C in a 5% CO_2_/95% air-humidified incubator for 24 hours following stimulation with immobilized anti-CD3 mAb (OKT3; ATCC, Rockville, MD, USA), which had previously been coated onto the six-well culture plates at 10 μg/ml overnight at 4°C.

Alternatively, T cells were presented with different saturating concentrations of the following: 25 ng/ml TNFα (gift from Dr W. Stec, Centre of Macromolecular Studies, Lodz, Poland), 100 ng/ml IL-6 (gift from Dr P. Ramage, Sandoz, Pharma Ltd., Basel, Switzerland) and 25 ng/ml IL-2 (gift from Dr U Gubler, Hoffmann-LaRoche, Nutley, NJ) for 8 days in culture, prior to fixation.

In all instances, control T cells were cultured in the absence of any stimulus. Following stimulation, T cells were harvested and washed three times in RPMI 1640 prior to fixation for 1 minute in PBS containing 0.05% glutaraldehyde, and were than neutralized with an equivalent volume of T-cell neutralizing buffer containing 0.2 M glycine. Following a further three washes the fixed T cells were resuspended in complete medium (RPMI 1640 containing 5% heat-inactivated FCS) at 2 × 10^6 ^cell/ml and stored for up to 7 days at 4°C until use. The T cells were washed twice in complete medium prior to use.

### Isolation of CD3^+ ^cells from synovial membrane tissue

Mononuclear cells were obtained from synovial tissue specimens taken during joint replacement surgery, provided by the Orthopedic/Plastic Surgery Department of Charing Cross Hospital, London, UK. Tissue was teased into small pieces and digested in medium containing 0.15 mg/ml DNAase type I (Sigma, Gillingham, Dorset, UK) and 5 mg/ml collagenase (Roche, Welwyn Garden City, Hertfordshire, UK) for 1–2 hours at 37°C. Cells are passed through a nylon mesh to exclude cell debris, washed and resuspended in RPMI (supplemented with 10% heat-inactivated FCS) at a density of 1 × 10^6 ^cells/ml. Mononuclear cells were incubated with anti-CD3 monoclonal antibody-coated Dynabeads for 20 minutes at 4°C under constant rotation. Cells attached to beads were isolated using a magnetic particle concentrator (Dynal, Merseyside, UK) and cultured for 6 hours at 37°C. Detached cells were then removed from the magnetic beads and washed using the magnetic particle concentrator, which allows for isolation of CD3^+ ^cells yielding high purity (>99%) and high viability (>95%). Cells were then fixed using the same protocol described above.

### Adenoviral vectors and their propagation

Adenoviral gene transfer is a technique used for efficient gene transfer into dividing and nondividing cells, such as fibroblasts and monocytes [24,25]. Recombinant replication-deficient adenoviral vector containing no insert (Adv0) was provided by M. Wood (University of Oxford, UK), and the adenovirus encoding porcine IκBα with a cytomegalovirus promoter and nuclear localization sequence (AdvIκBα) [26] was provided by Dr R. deMartin (Vienna, Austria). Briefly, viruses were propagated in the 293 human embryonic kidney cell line and purified by ultracentrifugation through two caesium chloride gradients. Titres of viral stocks were determined by plaque assay in 293 cells after exposure to virus for 2 hours in serum-free RPMI 1640, followed by washing and re-culturing the cells in complete medium for 48–72 hours [27].

### Gene transfer into macrophage-colony stimulating factor-treated monocytes with adenovirus

Prior to adenoviral infection, freshly elutriated monocytes were cultured in a 175 cm^3 ^culture flask (Falcon) for 2 days in RPMI 1640 supplemented with 5% heat-inactivated FCS (complete medium) with 50 ng/ml macrophage-colony stimulating factor (M-CSF). This process upregulates the α_v_β_5 _integrin, which acts as a cofactor for adenovirus infection [28,29]. Following culture, M-CSF-differentiated monocytes were washed once with PBS to remove nonadherent cells and the remaining adherent monocytes were incubated with 10 ml cell dissociation solution (Sigma) for 30–45 minutes until removed from the plastic. The cell suspension was washed three times in complete medium and the cell viability was assessed by trypan blue exclusion (>90%). Cells were plated at 2 × 10^5^/ml in 96-well flat-bottomed culture plates (Falcon) and were allowed to adhere for 1 hour prior to infection with adenovirus. The media and nonadherent cells were removed from each well and replaced with serum-free RPMI 1640 and adenovirus at the required multiplicity of infection (MOI) for 2 hours. Following incubation, the medium was removed and replaced with complete medium. Monocytes were cultured for a further 2 days before stimulation to enable adenoviral production of IκBα to reach optimal levels.

### Coculture of M-CSF-differentiated macrophages and lymphocytes

In the assays for contact-dependent chemokine production, M-CSF-differentiated monocytes (with or without IκBα transduction) were replated at 1 × 10^5 ^cells per well on a flat-bottom 96-well plate. Fixed lymphocytes were then added to the wells to give a final T cell:monocyte ratio of 7:1 and a final assay volume of 200 μl. Cultures containing monocytes alone and cultures containing lymphocytes alone were also included as experimental controls. Further controls included cocultures containing a porous membrane insert to physically separate the two populations, while allowing the transition of soluble mediators (0.2 μm Anopore^® ^Membrane Nunc Tissue Culture Inserts; Nunc, Roskilde, Denmark). After 18 hours of culture at 37°C (5% CO2, humidified atmosphere), the supernatants were harvested and stored at -70°C for subsequent chemokine assay.

### Measurement of chemokines by sandwich ELISA

Concentrations of IL-8 (CXCL8) (PharMingen, San Diego, CA, USA), GROα (CXCL1), interferon-gamma-inducible protein 10 (IP-10) (CXCL10), MCP-1 (CCL2), MIP-1α (CCL3), MIP-1β (CCL4) and RANTES (CCL5) were determined by ELISA (R&D Systems, **Oxford**, UK), following the manufacturer's instructions. The absorbance was read and analysed at 450 nm on a spectrophotometric ELISA plate reader (Labsystems Multiskan Biochromic, Labsystems, Uxbridge, UK) using the Delta soft II.4 software programme (DeltaSoft Inc, Hillsborough, NJ, USA). Results are expressed as the mean concentration of triplicate cultures ± standard deviation.

### Statistical analysis

Results were examined for statistical differences using Student's *t *test (two-tailed). *P *< 0.05 was considered significant, and such values are illustrated on the figures as appropriate.

## Results

### Both Tck cells and Ttcr cells induce contact-dependent chemokine production by M-CSF-differentiated human monocytes

**Figure 1:**
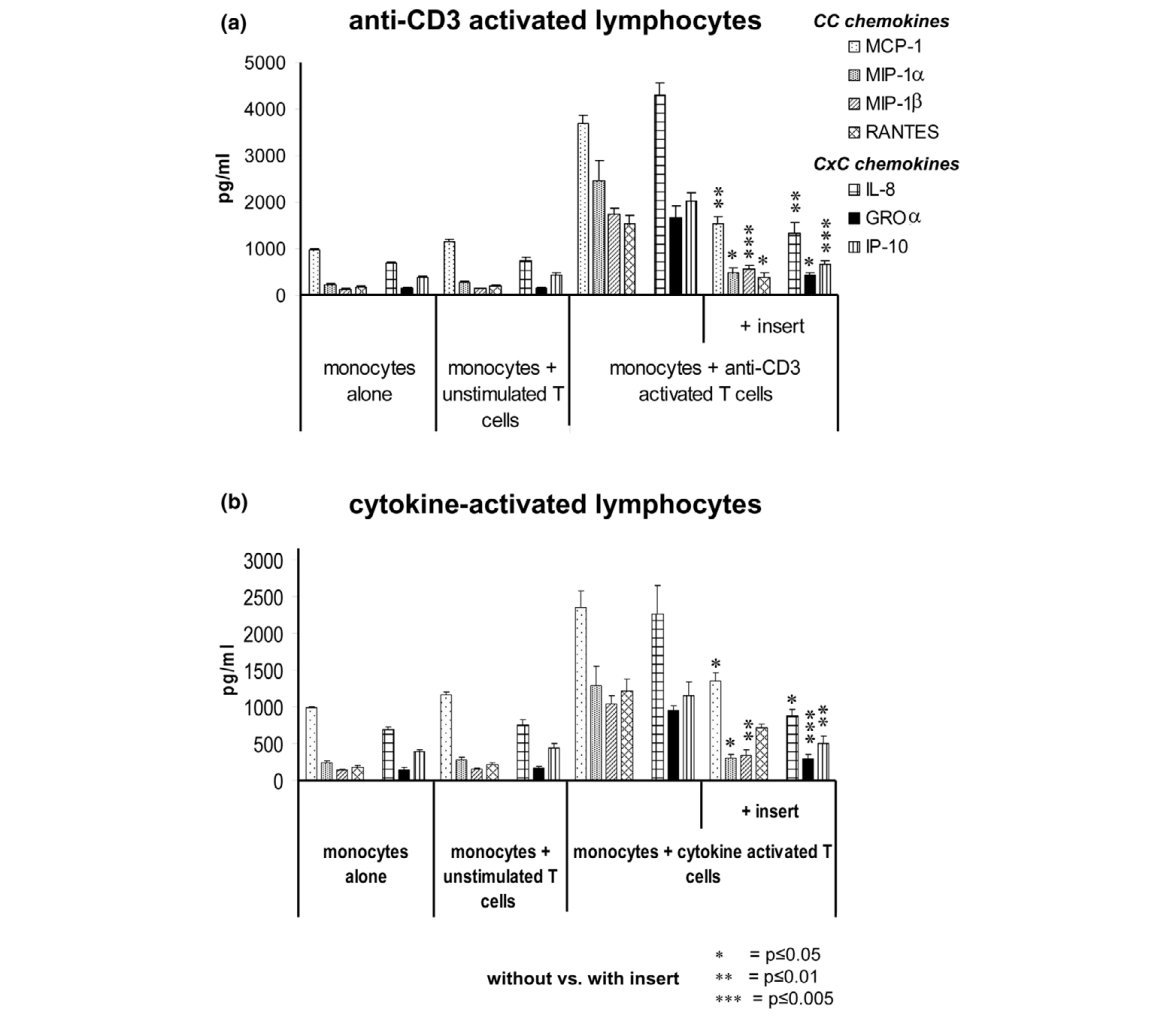
Activated T cells induce contact-dependent chemokine production by human macrophages. Lymphocytes were left unstimulated or were stimulated with either anti-CD3 for 48 hours (Ttcr cells) or a 'cocktail' of inflammatory cytokines (tumour necrosis factor alpha (TNFα), IL-2, IL-6) (Tck cells) for 8 days, before fixation. The unstimulated, Ttcr and Tck populations were then cultured with macrophage-colony stimulating factor-differentiated monocytes (ratio 7:1) for 18 hours. Culture supernatants were then isolated and levels of CC chemokines (monocyte chemoattractant protein 1 (MCP-1), macrophage inflammatory protein 1 alpha (MIP-1α), macrophage inflammatory protein 1 beta (MIP-1β), RANTES) and CXC chemokines (IL-8, growth-related gene product alpha (GROα) and interferon-gamma-inducible protein (IP-10)) measured by ELISA. In some cases, a porous membrane insert was used to physically separate the two populations, while allowing the transition of soluble mediators. Results are shown from **(a) **Ttcr-cell lymphocyte cultures and **(b) **Tck-cell lymphocyte cultures. Data represent a mean of triplicate cultures ± standard deviation and are representative of at least three experiments. Statistically significant differences in chemokine detection are indicated.

We have previously reported that the production of proinflammatory cytokines by macrophages can be induced by cognate interaction with Ttcr cells or Tck cells [19,21]. In the present paper we investigated whether activated T cells can also induce macrophage CC or CXC chemokine secretion in a contact-dependent manner. We found that, upon coculture, T cells activated with anti-CD3 antibody are able to induce production of high levels of chemokines in M-CSF-differentiated human monocytes (macrophages). Levels of both CC chemokines (MCP-1, MIP-1α, MIP-1β and RANTES) and CXC chemokines (IL-8, GROα and IP-10) are all elevated in comparison with those found in cultures of M-CSF-differentiated monocytes alone (Figure 1a). This induction of chemokine production in monocytes can be significantly reduced if the monocytes and T cells are physically separated using a porous membrane insert, demonstrating the importance of cell-cell contact in the induction process. In contrast, chemokine production by M-CSF-differentiated monocytes alone remains unchanged following coculture with unstimulated T cells (cultured for 24 hours prior to fixation).

Tck cells were also cultured with M-CSF-differentiated monocytes (Figure 1b). Tck cells, as seen with Ttcr cells, were able to induce significant production of all CC chemokines (MCP-1, MIP-1α, MIP 1β and RANTES) and CXC chemokines (IL-8, GROα and IP-10) assayed to similar levels, again in a contact-dependent manner. As expected, fixed Ttcr and Tck cells cultured alone did not secrete any detectable levels of chemokines (data not shown). Moreover, macrophages cultured in the presence of the insert and stimulated with lipopolysaccharide (LPS) secreted high levels of chemokines (data not shown) as previously described [30], indicating that the presence of the membrane insert does not influence macrophage function.

### Differential utilization of NFκB in the Ttcr-cell and Tck-cell contact-dependent induction of CC and CXC chemokines in M-CSF-differentiated monocytes

We have previously shown that the contact-dependent induction of TNFα production in resting monocytes by Tck cells or RA synovial T cells is abrogated by blockade of the transcription factor NFκB [23]. As NFκB is a major transcription factor regulating the expression of numerous genes involved in immune and inflammatory responses [28,31], we determined whether T-cell contact-dependent production of chemokines is also regulated by NFκB.

To inhibit NFκB with specificity we employed an efficient adenoviral gene transfer method to overexpress IκBα in human macrophages. We have previously shown that high levels of IκBα are achieved by AdvIκBα transduction that remain elevated even after LPS stimulation [30]. As IκBα is a major inhibitory component of the NFκB pathway, increased expression of IκBα blocks NFκB nuclear translocation and DNA binding induced by LPS.

**Figure 2:**
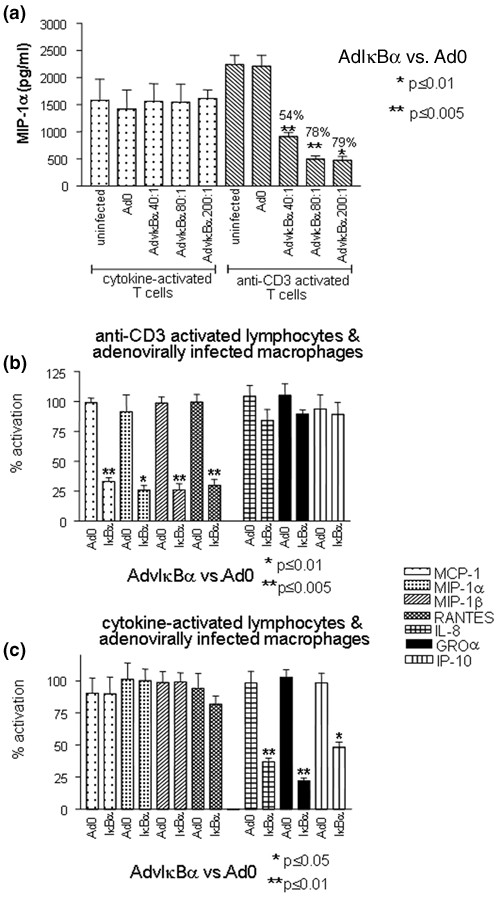
Differential utilization of NFκB in activated-T-cell contact-dependent chemokine production by human macrophages. Macrophage-colony stimulating factor-differentiated monocytes were infected with AdIκBα or Ad0, an empty control virus. After a further 2 days of culture and replating, anti-CD3-activated T cells (Ttcr cells) and cytokine-activated T cells (Tck cells) were added at a lymphocyte:monocyte ratio of 7:1. After 18 hours, culture supernatants were isolated and levels of CC chemokines (monocyte chemoattractant protein 1 (MCP-1), macrophage inflammatory protein 1 alpha (MIP-1α), macrophage inflammatory protein 1 beta (MIP-1β), RANTES) and CXC chemokines (IL-8, growth-related gene product alpha (GROα) and interferon-gamma-inducible protein (IP-10)) were measured simultaneously by ELISA. **(a) **MIP-1α levels in uninfected, Ad0-infected (multiplicity of infection (MOI) 200:1) and AdIκBα-infected (MOI 40:1, 80:1 and 200:1) monocyte cultures when stimulated with Ttcr-cells or Tck-cells. **(b) **and **(c) **Levels of CC and CXC chemokines in Ad0-infected and AdIκBα-infected monocytes (MOI 80:1) following stimulation with (b) Ttcr cells and (c) Tck cells. Data represent the mean of triplicate cultures ± standard deviation and are representative of at least three experiments. Statistically significant reduction in chemokine levels in AdvIκBα-infected (as compared with Ad0-infected) cultures is indicated.

We then examined whether IκBα overexpression inhibits monocyte chemokine production induced by Ttcr cells. We found that AdIκBα inhibits the production of CC chemokines induced by contact with Ttcr cells but has no effect on CXC chemokine induction. MIP-1α production induced by Ttcr cells was therefore profoundly reduced, in a dose-dependent manner, in M-CSF-differentiated monocytes infected with AdIκBα but not with Ad0, a control virus without insert. At MOI of 40:1 and 80:1, the inhibition of MIP-1α expression was 54% (*P *≤ 0.005) and 78% (*P *≤ 0.005), respectively – which was not further increased at higher MOI (Figure 2a).

Similar significant inhibition of the production of the other CC chemokines MIP1-β (73.9%, *P *≤ 0.005), RANTES (70.2%, *P *≤ 0.005) and MCP-1 (67%, *P *≤ 0.005) was also observed in AdIκBα-infected monocytes (Figure 2b). In contrast, there was no effect of IκBα overexpression on CXC chemokine production. We found that there was no significant inhibition of the chemokines GROα, I,L-8 or IP-10 in AdIκBα-infected monocytes activated by Ttcr cells, suggesting that there is differential utilization of NFκB for the expression of CC and CXC chemokines in this system.

We also examined the role of NFκB in the Tck-cell contact-dependent production of chemokines in monocytes. Unexpectedly, we found that IκBα overexpression inhibited Tck-cell-dependent CXC chemokine production in M-CSF-differentiated monocytes, but had no effect in CC chemokine production. Thus, although contact-dependent induction of GROα, IL-8 and IP-10 was significantly inhibited in AdIκBα-infected macrophages by 78.7% (*P *≤ 0.01), 63.2% (*P *≤ 0.01) and 52.1% (*P *≤ 0.05), respectively, the induction of MIP-1α, MIP-1β, RANTES and MCP-1 was unaffected (Figure 2c). This inverted pattern of utilization of NFκB for the T-cell contact-dependent induction of CC and CXC chemokines in monocytes is surprising and indicates that chemokine gene expression may be more complex than previously thought.

### IκBα overexpression significantly inhibits rheumatoid T-cell-induced macrophage secretion of CXC, but not CC, chemokines

**Figure 3:**
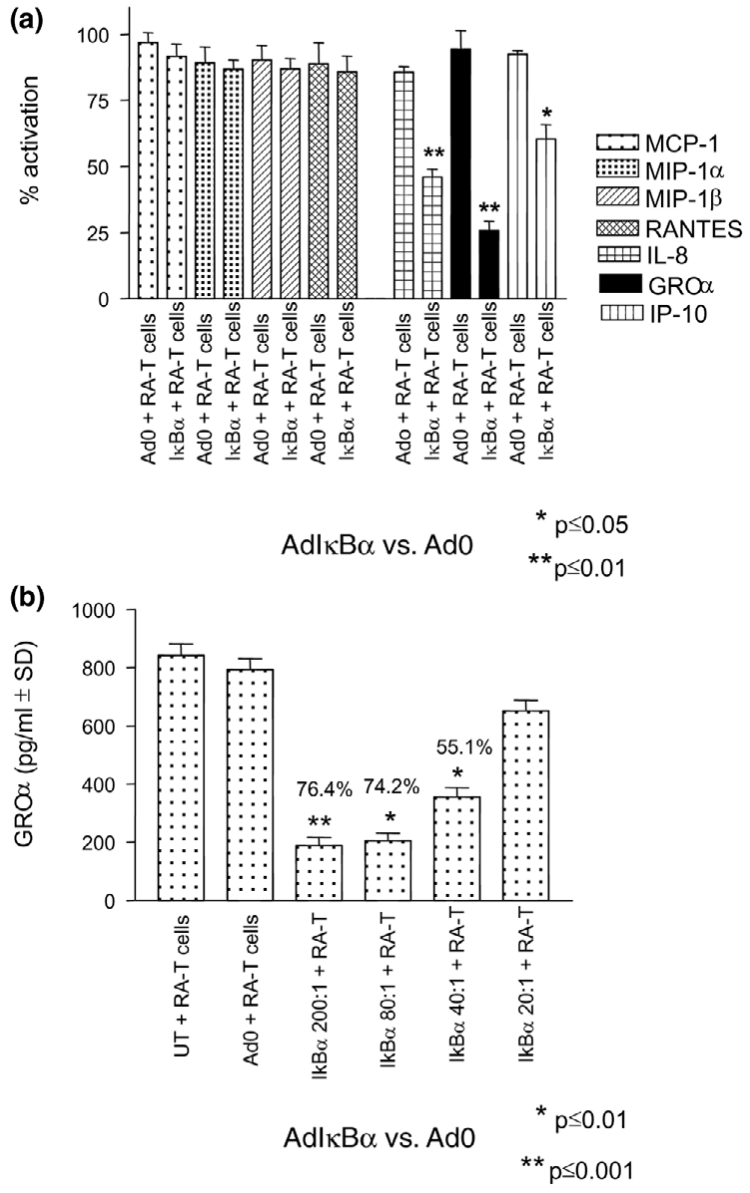
IκBα overexpression significantly inhibits rheumatoid T-cell-induced macrophage chemokine secretion of CXC, but not CC, chemokines. Using anti-CD3 labelled Dynabeads, synovial T cells were enriched from the mixed cell population obtained following enzymatic dissociation of synovial tissue samples from rheumatoid arthritis (RA) patients. Fixed RA T cells were cultured with macrophage-colony stimulating factor-differentiated monocytes infected with Ad0 and AdvIκBα at a T cell:monocyte ratio of 7:1 as described in Figure 2. After 18 hours, culture supernatants were isolated and levels of CC chemokines (monocyte chemoattractant protein 1 (MCP-1), macrophage inflammatory protein 1 alpha (MIP-1α), macrophage inflammatory protein 1 beta (MIP-1β), RANTES) and CXC chemokines (IL-8, growth-related gene product alpha (GROα) and interferon-gamma-inducible protein (IP-10)) were measured by ELISA. **(a) **Levels of chemokines for monocytes infected with Ad0 and AdIκBα (multiplicity of infection (MOI) 80:1) following stimulation with RA T cells. **(b) **GROα levels in uninfected, Ad0-infected (MOI 200:1) and AdvIκBα-infected (MOI 20:1, 40, 80:1 and 200:1) monocyte cultures when stimulated with RA T cells. Data represent the mean of triplicate cultures ± standard deviation and are representative of at least three experiments. Statistically significant reduction in chemokine levels in AdIκBα-infected (as compared with Ad0-infected) cultures is indicated.

We next investigated whether RA synovial T cells enriched from dissociated RA synovial tissue could also induce monocyte chemokine secretion in a contact-dependent manner and whether this requires NFκB. RA synovial T cells were isolated from dissociated synovial membranes using anti-CD3 DynaBeads, as described in Materials and methods. We found that, like Ttcr and Tck cells, fixed RA synovial T cells were able to induce both CC and CXC chemokine production from M-CSF-differentiated human monocytes (Figure 3). Furthermore, overexpression of IκBα in these monocytes resulted in impaired RA synovial T cell-dependent CXC chemokine release, when compared with Ad0-infected monocytes. A significant reduction in IL-8 (54.1%, *P *≤ 0.01), IP-10 (39.6%, *P *≤ 0.05) and GROα (74.2%, *P *≤ 0.01) production was therefore observed (Figure 3a). This effect was dose dependent, with increasing MOI of 40:1 and 80:1 inducing a reduction in GROα levels of 55.1% (*P *≤ 0.01) and an optimal 74.2% (*P *≤ 0.001), respectively (Figure 3b).

Similar dose-dependent profiles were observed for the other chemokines tested (data not shown). Interestingly, however, overexpression of IκBα had no significant effect on the expression of CC chemokines by M-CSF-differentiated monocytes, suggesting that RA synovial T cells possess similarities in their effector function to Tck cells, rather than Ttcr cells. It is noteworthy that RA T cells isolated based on CD2 expression have previously demonstrated an identical effector function to those isolated using anti-CD3 (data not shown), thus discounting the idea that CD3-based methods may influence the behaviour of RA T cells (through the potential for crosslinking) in this system.

### The phosphatidyl-inositol-3-kinase pathway regulates both NFκB-dependent and NFκB-independent contact-mediated chemokine production

**Figure 4:**
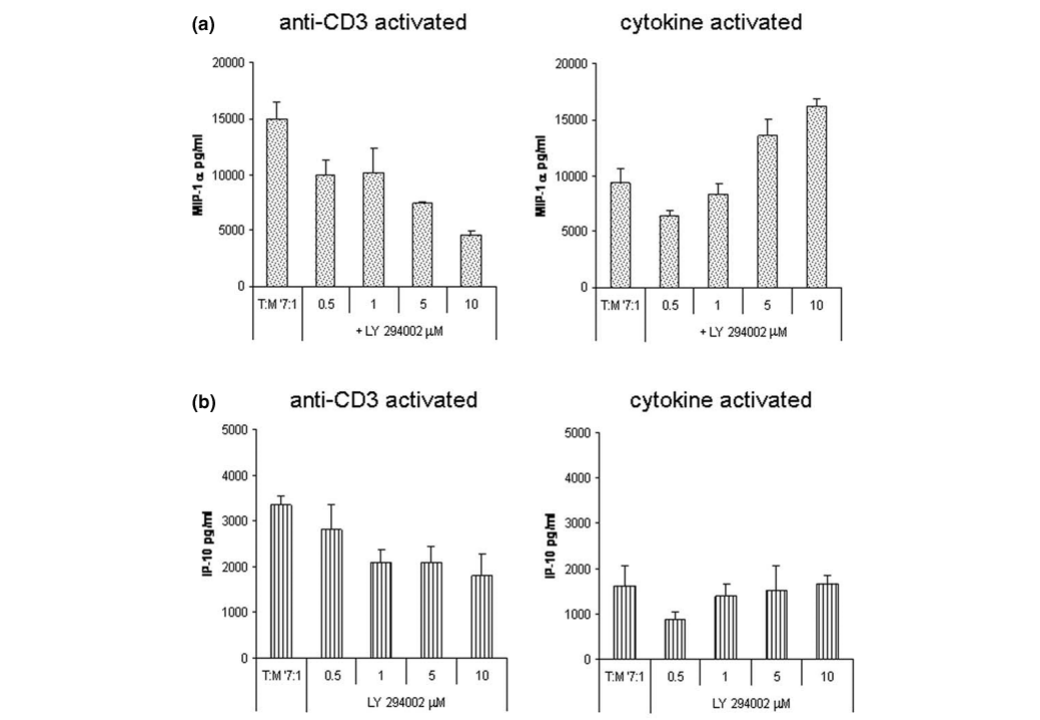
The phosphatidyl-inositol-3-kinase pathway regulates both NFκB-dependent and NFκB-independent contact-dependent chemokine production. Macrophage-colony stimulating factor-differentiated monocytes were preincubated for 30 minutes in the presence or absence of variable amounts of LY294002 (as shown) before being stimulated with anti-CD3-activated T cells (Ttcr) or cytokine-activated T cells (Tck) at a T cell:monocyte ratio of 7:1. After 18 hours, culture supernatants were isolated and levels of **(a) **macrophage inflammatory protein 1 alpha (MIP-1α) (CC chemokine) and **(b) **interferon-gamma-inducible protein (IP-10) (CXC chemokine) were measured by ELISA. Data represent the mean of triplicate cultures ± standard deviation and are representative of at least three experiments.

Finally, we investigated what further cell signalling pathways (in addition to NFκB) could play a potential role in contact-dependent chemokine production. Ttcr cells and Tck cells were used to stimulate monocytes that had been pretreated with a chemical inhibitor of the PI3K pathway (LY294002), and the resulting effects on chemokine production were determined. We found that Ttcr-induced IP-10 (CXC chemokine) production (NFκB independent) was dose-dependently reduced in the presence of the inhibitor (Figure 4b). In contrast, Tck-induced MIP-1α (CC chemokine) production (also NFκB independent) could be dose-dependently enhanced in the presence of the PI3K inhibitor (Figure 4a), indicating the pathway plays a positive and negative regulatory role in each respective case. With NFκB-dependent Ttcr-induced MIP-1α production also displaying PI3K dependence, however, a role for this pathway in NFκB-dependent as well as NFκB-independent chemokine production cannot be ruled out.

## Discussion

We have previously shown that TNFα synthesis in RA synovial cultures is T-cell contact-dependent; T-cell depletion or physical separation from the rest of the cells rapidly diminished macrophage TNFα production in these cultures [23]. We have also shown that the contact-dependent effector function of RA T cells in the joint resembles that displayed by Tck cells, which can be expanded from normal blood with cytokines found in the RA joint and in the absence of TCR engagement [21,23]. Both RA synovial T cells without further activation and Tck cells induced TNFα production in resting monocytes in a cell-contact dependent manner, which was abrogated by blockade of the transcription factor NFκB but was augmented if PI3K was inhibited. Normal blood T cells activated 'conventionally' via the TCR with cross-linked anti-CD3 antibody (Ttcr cells) do not reproduce this effector function of RA T cells [23]. In this study, we investigated whether Tck cells or RA synovial T cells also regulate chemokine production from macrophages and whether this was mediated in a contact-dependent manner.

Using a coculture system consisting of fixed lymphocytes and M-CSF-differentiated human monocytes [21,23], we demonstrate in this manuscript that Tck cells stimulate monocytes to secrete high levels of several CC and CXC chemokines that include MIP-1α, MIP-1β, RANTES, MCP-1, GROα, IL-8 and IP-10. This was a T-cell contact-dependent process as the physical separation of T cells from monocytes through the use of a transwell insert abrogated this effect. This observation was also true for Ttcr cells and RA synovial T cells but not for control nonactivated T cells, suggesting that contact-dependent regulation of macrophage chemokine production is a general property of activated T cells. Several other groups have also shown the importance of T-cell contact in regulating the production of cytokines and tissue destructive enzymes (such as matrix metalloproteinases) by monocyte/macrophages [15,17-21,32] and fibroblasts, suggesting that this may be a major mechanism of promoting inflammation in chronic inflammatory diseases where there is an absence of infection or infectious agents [33].

Various stimuli induce T cells to activate monocytes/macrophages by cellular contact, including anti-CD3 cross-linking with or without anti-CD28 stimulation (as used in this study) [34], cytokines such as IL-2, IL-6 and TNFα (as used in this study) [19] or IL-15 [15], phytohaemagluttinin/phorbol myristate acetate [17,35,36] and antigen recognition on antigen-specific T-cell clones of the Th1 or Th2 phenotype [37,38]. Depending on the T-cell type and the stimulus used, the pattern of gene expression triggered in monocytes/macrophages by T-cell contact differs. We have previously shown that although Ttcr cells activate monocytes to produce both TNFα and IL-10, Tck cells only trigger the production of TNFα in monocytes, suggesting that this is a mechanism by which the cytokine balance is skewed towards the proinflammatory side in RA [19]. Other studies have shown that Th1 clones preferentially induce IL-1β rather than IL-1 receptor antagonist over other T-cell clones [38,39]. This suggests that multiple ligands and counter-ligands are involved in the contact-mediated activation of monocytes/macrophages that are differentially induced on T cells (depending on the stimulus) and differentially induce monocyte/macrophage signal transduction.

The transcription factor NFκB has been shown to regulate both inflammatory and tissue destructive processes in RA [25,40]. Many of the promoter regions of chemokines are known to have κB sites in their promoters and include IL-8 [41], GROα [42], IP-10 [43], MCP-1 [44], and RANTES [45]. We recently used adenoviral gene transfer of IκBα to block NFκB in human M-CSF-differentiated monocytes, and showed that the expression of CC chemokines MIP-1α, MCP-1 and RANTES induced by TNFα or LPS was NFκB dependent, as was the expression of CXC chemokines IL-8, GROα and epithelial neutrophil activating peptide 78 induced by TNFα [30]. The expression of these CXC chemokines induced by LPS, however, was found to be NFκB independent – indicating that the requirement for this transcription factor in the regulation of chemokine gene expression is complex and dependent on the stimuli used.

In this study, we used the same system of adenovirally mediated IκBα overexpression in M-CSF-differentiated monocytes to investigate the potential involvement of NFκB in the expression of CC and CXC chemokines induced by contact with activated T cells or RA synovial T cells. Surprisingly, we found that blocking NFκB resulted in differential inhibition of CC and CXC chemokines depending on whether Ttcr cells, Tck cells or rheumatoid T cells were used to stimulate M-CSF-differentiated monocytes. CC chemokine production was thus found to be NFκB dependent when mediated by Ttcr cells, but NFκB independent when mediated by Tck or RA synovial T cells. In addition, CXC chemokine production was found to be NFκB independent when mediated by Ttcr cells, but largely NFκB dependent when mediated by Tck or RA synovial T cells. These data suggest that, through different molecular interactions, at least two differential pathways of monocyte chemokine production are induced by Ttcr cells and Tck cells that differ in the rate-limiting involvement of NFκB. Evidence from our inhibitor studies suggest involvement of the PI3K pathway in regulating both NFκB-independent and NFκB-dependent chemokine production, in either a positive or negative manner, depending on chemokine and lymphocyte stimulus. We have previously published work showing a similar augmentation of Tck/RA T-cell-induced TNFα production in the presence of these inhibitors [23].

As the promoters of all the chemokines studied here contain NFκB binding sites, this raises the obvious question of how this effect is regulated. Currently unclear is whether these specific sites are functioning as positive or negative regulators of transcription; a process that could itself be influenced by which other pathways are also activated. For example, TNFα production in T cells is known to be regulated by nuclear factor of activated T cells although the TNF gene contains at least five NFκB sites [46]. Furthermore, variable factors such as the site sequence and its distance from the transcription start site, as well as the nature of the different NFκB dimers recruited to the site, will all interact to influence gene expression [47].

A further layer of complexity operating in this system is the role of contact-induced TNFα in secondary chemokine production. We have previously shown TNFα production itself is differently dependent on NFκB and the PI3K pathway (similarly regulating either positively or negatively) according to Ttcr-cell or Tck-cell induction processes. As such, effects on both pathways could be acting on chemokine induction in direct and indirect ways. Furthermore, our previous studies have shown both Ttcr-cell-induced and Tck-cell-induced TNFα production to be p38MAPK dependent, but p42/p44 MAPK independent (data not shown), indicating that mitogen-activated protein kinases may also be involved in contact-dependent chemokine induction.

## Conclusion

This study demonstrates for the first time that RA synovial T cells as well as Tck cells are able to induce monocyte chemokine production in a contact-dependent manner and through NFκB-dependent and NFκB-independent mechanisms, in a process influenced by the PI3K pathway. In addition, these data provide further evidence that Tck cells share aspects of their effector function (such as contact-mediated monocyte chemokine production) with RA synovial T cells. Furthermore, these data demonstrate one more function of RA T cells; namely, their ability to induce monocyte/macrophage chemokine secretion by cellular contact. The observation that RA synovial T cells mirror the behaviour of cytokine-driven, rather than CD3-activated, cells is consistent with the notion that antigen-independent responses play a key role in RA. As such, this study further emphasizes that T cells are not simply 'innocent bystanders' in RA, but can be important drivers of chronic inflammation through antigen-independent mechanisms [48,49].

## Abbreviations

ELISA = enzyme-linked immunosorbent assay; FCS = foetal calf serum; GROα = growth-related gene product alpha; IL = interleukin; IP-10 = interferon-gamma-inducible protein 10; LPS = lipopolysaccharide; mAb = monoclonal antibody; MCP-1 = monocyte chemoattractant protein 1; M-CSF = macrophage-colony stimulating factor; MIP-1α = macrophage inflammatory protein 1 alpha; MIP-1β = macrophage inflammatory protein 1 beta; MOI = multiplicity of infection; NFκB = nuclear factor kappa B; PBS = phosphate-buffered saline; PI3K = phosphatidyl-inositol-3-kinase; RA = rheumatoid arthritis; Tck cells = cytokine-activated T cells; TCR = T-cell receptor; Ttcr cells = anti-CD3-activated T cells; TNFα = tumour necrosis factor alpha.

## Competing interests

The authors declare that they have no competing interests.

## Authors' contributions

JTB participated in data analysis, assembly and creation of the figures, and manuscript writing. EA contributed to the study design, experimentation, data analysis, assembly and creation of the figures, and manuscript writing. CJC was involved in the study design, experimentation and data analysis. PG contributed to the study design, experimentation, data analysis, and assembly and creation of the figures. BMJF and FMB were responsible for the initiation of the study, review of the analysed data and manuscript writing.
